# Cadmium Exposure and Neurodevelopmental Outcomes in U.S. Children

**DOI:** 10.1289/ehp.1104152

**Published:** 2012-01-27

**Authors:** Timothy Ciesielski, Jennifer Weuve, David C. Bellinger, Joel Schwartz, Bruce Lanphear, Robert O. Wright

**Affiliations:** 1 Department of Environmental Health, Harvard School of Public Health, Boston, Massachusetts, USA; 2 Rush Institute for Healthy Aging, Rush University Medical Center, Chicago, Illinois, USA; 3 Department of Neurology, Children’s Hospital Boston, Boston, Massachusetts, USA; 4 Harvard Medical School, Boston, Massachusetts, USA; 5 Cincinnati Children’s Environmental Health Center, Cincinnati Children’s Hospital Medical Center, Cincinnati, Ohio, USA; 6 Child and Family Research Institute, BC Children’s Hospital and Faculty of Health Sciences, Simon Fraser University, Vancouver, British Columbia, Canada; 7 Department of Pediatrics, Children’s Hospital, Boston, Massachusetts, USA

**Keywords:** attention deficit hyperactivity disorder, cadmium, learning disability, neurodevelopment, neuropsychological development, NHANES, risk assessment, special education

## Abstract

Background: Low-level environmental cadmium exposure in children may be associated with adverse neurodevelopmental outcomes.

Objective: Our aim was to evaluate associations between urinary cadmium concentration and reported learning disability (LD), special education utilization, and attention deficit hyperactivity disorder (ADHD) in U.S. children using National Health and Nutrition Examination Survey (NHANES) data.

Methods: We analyzed data from a subset of participants in NHANES (1999–2004) who were 6–15 years of age and had spot urine samples analyzed for cadmium. Outcomes were assessed by parent or proxy-respondent report. We fit multivariable-adjusted logistic regression models to estimate associations between urinary cadmium and the outcomes.

Results: When we compared children in the highest quartile of urinary cadmium with those in the lowest quartile, odds ratios adjusted for several potential confounders were 3.21 [95% confidence interval (CI): 1.43, 7.17] for LD, 3.00 (95% CI: 1.12, 8.01) for special education, and 0.67 (95% CI: 0.28, 1.61) for ADHD. There were no significant interactions with sex, but associations with LD and special education were somewhat stronger in males, and the trend in the ADHD analysis was only evident among those with blood lead levels above the median.

Conclusions: These findings suggest that children who have higher urinary cadmium concentrations may have increased risk of both LD and special education. Importantly, we observed these associations at exposure levels that were previously considered to be without adverse effects, and these levels are common among U.S. children.

Cadmium is a heavy metal found in the earth’s crust that is disseminated in the environment both by natural processes and by human activities such as fossil fuel burning, waste incineration, smelting procedures, and the use of phosphate fertilizers (Agency for Toxic Substances and Disease Registry 2008). Uptake of environmental cadmium in plants and animals results in human exposures via food or tobacco smoke, and occupational exposures can occur as well [European Food Safety Authority (EFSA) 2009]. Cadmium is known to be nephrotoxic (EFSA 2009), but there is also a growing body of evidence suggesting that cadmium exposure may have adverse neurodevelopmental consequences.

Several animal studies have reported effects of cadmium on electrophysiological parameters, markers of neurotransmitter function, and neurobehavioral outcomes (Ali et al. 1986; Desi et al. 1998; Lehotzky et al. 1990; Nagymajtenyi et al. 1997; Nation et al. 1983, 1984, 1989, 1990). Studies in children have reported associations between higher cadmium levels and mental retardation (Jiang et al. 1990; Marlowe et al. 1983), decreased verbal IQ (Thatcher et al. 1982), lower neuropsychological test performance (Bonithon-Kopp et al. 1986; Stellern et al. 1983), learning disability (LD) (Capel et al. 1981; Ely et al. 1981; Pihl and Parkes 1977), poor reading performance (Thatcher et al. 1984b), neurophysiological evoked potential differences (Thatcher et al. 1984a), and behavioral problems in the presence of concurrently elevated lead levels (Marlowe et al. 1985a). In contrast, other human studies have failed to detect significant multivariable-adjusted associations between markers of cadmium exposure and neurodevelopmental outcomes (Cao et al. 2009; Gillberg et al. 1982; Lee et al. 2007; Marlowe et al. 1985b; Moon et al. 1985; Wright et al. 2006). These studies varied in size, quality, and design. In addition, they used several different exposure metrics, evaluated different windows of susceptibility, and differed in their consideration of potentially important confounders. These factors may help explain the inconsistent results, and further research could help resolve some of the discrepancies.

In this study, we analyzed a large representative sample of U.S. children 6–15 years of age from the National Health and Nutrition Examination Survey (NHANES), to determine whether higher levels of urinary cadmium were associated with attention deficit hyperactivity disorder (ADHD), LD, or placement in special education. To our knowledge, this is the largest study to evaluate associations between urinary cadmium and neurodevelopmental outcomes, and the first to do so in a nationally representative sample of U.S. children.

## Methods

*Data source and study population.* NHANES is an ongoing series of cross-sectional questionnaires, examinations, and laboratory analyses that evaluate nutritional and environmental exposures as well as various health parameters in the U.S. population [Centers for Disease Control and Prevention National Center for Health Statistics (CDC-NCHS) 2010b]. Detailed documentation on NHANES is available online (CDC-NCHS 2010b). For this study we used NHANES data collected 1999–2004. We restricted our analyses to children 6–15 years of age because urinary cadmium was evaluated in a subsample of participants ≥ 6 years of age, and information on several covariates was not available for participants > 15 years of age. There were 2,282 children between 6 and 15 years of age in this subsample, and urine cadmium measurements were available for 2,199 (96.4%) of these. Because some children were missing outcome data, 2,189, 2,196, and 2,195 children were included in the analyses of LD, special education, and ADHD, respectively. The NHANES website notes that approval (protocol 98-12) was obtained from the NCHS Ethics Review Board (referred to as the NHANES Institutional Review Board before 2003) (CDC-NCHS 2012).

*Exposure assessment.* Cadmium exposure was assessed using urinary cadmium concentration, which is an indicator of body burden/cumulative cadmium exposure (Lauwerys et al. 1994). Urinary cadmium concentration was determined by inductively coupled plasma mass spectrometry (ICP-MS), and details are accessible online via the NHANES website (CDC-NCHS 2010b). The limit of detection (LOD) was 0.06 μg/L, and cadmium concentrations below the LOD were imputed as the LOD divided by the square root of 2. Of the 2,199 participants, 222 (10%) had urinary cadmium concentrations below the LOD. Cadmium (Cd) concentrations were corrected for interference from tin (Sn) and molybdenum (Mo), because these elements can produce ICP-MS signals (^114^Sn, ^98^Mo^16^O^+^, ^96^Mo^18^O^+^, ^97^Mo^17^OH^+^) that overlap with ^114^Cd (CDC-NCHS 2010b; Jarrett et al. 2008). Cadmium concentrations that were below zero after molybdenum correction were left-truncated and listed as zero.

Urinary creatinine is often used to correct for the variation in chemical concentration that is attributable to variation in urine dilution when exposure estimates are based on spot urine samples (Barr et al. 2005). Because creatinine enters urine at a fairly constant rate, urine creatinine concentration is inversely proportional to urine flow rate and can be used to correct for differences in urine flow rate (Barr et al. 2005). In this study urine creatinine levels were determined via a Jaffé rate reaction with a Beckman Synchron CX3 Clinical Analyzer (Beckman Instruments, Inc., Brea, CA, USA) (CDC-NCHS 2010b). As recommended by Barr et al. (2005), we included urine creatinine concentration as an independent covariate in the regression models rather than using the ratio of cadmium to creatinine (creatinine standardization), because this approach should be less likely to produce biased effect estimates (Barr et al. 2005; Schisterman et al. 2005).

*Outcomes.* Assessment of neurodevelopmental outcomes was based on responses of parent, guardian, or other adult proxy responder (CDC-NCHS 2010b). For children < 12 years of age, LD status was assessed with the following question: “Has a representative from a school or health professional ever told [you] that [the child] had a learning disability?”; ADHD status was assessed as follows: “Has a doctor or health professional ever told [you] that [the child] had attention deficit disorder?” For those ≥ 12 years of age, the same questions were asked about LD and ADHD but the bracketed words were replaced with: [the child] and [he/she]. Special education utilization was assessed with the same question regardless of the child’s age: “Does [the child] receive Special Education or Early Intervention Services?”

*Covariates.* Data on many potential confounders were available in NHANES (CDC-NCHS 2010b). We considered the following covariates in our analyses: age (years), sex, race/ethnicity [non-Hispanic white (referent), non-Hispanic black, Mexican American, other Hispanic, other race (including multiracial)], maternal age at birth of child (years), attendance at preschool/daycare (yes/no), health insurance coverage (yes/no), receipt of neonatal intensive care unit or special newborn care (yes/no), low birth weight (< 2,500 g; yes/no), poverty income ratio (the ratio of family income to the federal poverty threshold; ratios ≥ 5 are listed as 5), education level of the household reference person (highest grade achieved: less than 9th grade, at least 9th grade but no high school diploma, high school graduate/GED (general educational development) degree or equivalent, some college or associate of arts degree, and college graduate or above), blood lead level (micrograms per deciliter), low hemoglobin level for age and sex (< 10th percentile, ≥ 10th percentile; used as a proxy for iron deficiency), report of a smoker in the home (yes/no), serum cotinine (nanograms per milliliter), prenatal smoke exposure (i.e., the child’s mother smoked while pregnant; yes/no), and, as mentioned above, urine creatinine (milligrams per deciliter). Detailed information on the covariates is available online (CDC-NCHS 2010b).

*Statistical analysis.* Analyses were conducted using SAS (version 9.1.3; SAS Institute Inc., Cary, NC). We obtained national prevalence estimates for the outcomes by using the SURVEYMEANS procedure, specifying strata, cluster, and weight variables to account for the complex sampling design characteristics of NHANES (CDC-NCHS 2010a, 2010b). For each of the three outcomes, we estimated the relative odds of each outcome across quartiles of urinary cadmium concentration. To do this we fit multivariable-adjusted logistic regression models with the SURVEYLOGISTIC procedure, again specifying strata, cluster, and weight variables (CDC-NCHS 2010a, 2010b). Urinary cadmium was evaluated in quartiles in order to allow for the detection of nonlinear dose–response relationships, reduce the potential excess influence of data from children with very high cadmium concentrations on the effect estimates, and alleviate concerns about low cadmium levels in the context of molybdenum correction and left truncation of urinary cadmium levels at zero (when correction resulted in a cadmium concentration below zero).

We constructed three main models: *a*) logistic regression models relating urinary cadmium to each outcome adjusting only for urinary creatinine; *b*) core models adjusted for urinary creatinine, age, sex, blood lead, smoker in the home, serum cotinine, prenatal smoke exposure (mother smoked while pregnant), and poverty income ratio; and *c*) full models also adjusted for additional covariates of *a priori* interest that predicted outcomes (*p* < 0.10) in bivariate logistic regression models. Continuous and discrete covariates were modeled as single linear terms, except for hemoglobin levels. Low hemoglobin levels are a proxy for iron deficiency (Zimmermann 2008). Because normal hemoglobin levels vary by age and sex (Ahsan and Noether 2011), we modeled hemoglobin in categories (< 10th percentile or ≥ 10th percentile for the age and sex of the participant).

Prior studies have suggested an interaction between cadmium exposure and sex in relation to LD, as well as an interaction between cadmium exposure and lead exposure in relation to ADHD (Ely et al. 1981; Nation et al. 1989, 1990). We evaluated cadmium–sex and cadmium–lead interactions in the fully adjusted models by including product interaction terms with cadmium modeled as an ordinal trend variable (coded as 0, 1, 2, or 3 based on the quartile of exposure) to obtain *p*-values for each interaction. For the cadmium–lead interaction, lead was also modeled as an ordinal trend variable. To obtain a single (summary) effect estimate for urinary cadmium with each outcome, we evaluated this cadmium quartile trend variable in the fully adjusted models. We also evaluated this cadmium quartile trend variable in the fully adjusted models (with the sex or blood lead term removed) within strata defined by sex or blood lead level (above/below median). In our analyses statistical significance was defined as *p* < 0.05 or a 95% confidence interval (CI) that excluded the null.

## Results

**Figure 1:**
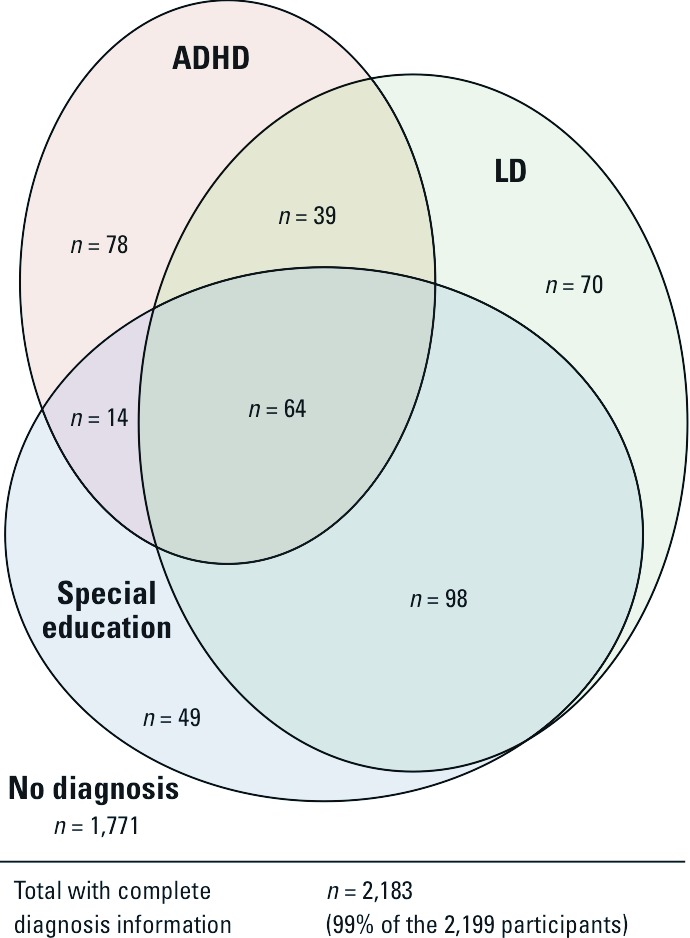
The co-occurrence of neurodevelopmental outcomes in the study population among the 2,183 participants with information on all three outcomes (99% of the 2,199 participants).

History of LD was reported for 12.6% (276 of 2,189) of the study population, and participation in special education was reported for 10.5% (231 of 2,196). History of ADHD diagnosis was reported for 9.0% (198 of 2,195) of the study population. The co-occurrence of the outcomes is shown in Figure 1. Accounting for the complex survey design characteristics of NHANES, the estimated lifetime prevalence among U.S. children 6–15 years of age was 12.6% for LD (95% CI: 9.5%, 15.7%), 11.7% for special education participation (95% CI: 9.5%, 13.9%), and 11.5% for ADHD (95% CI: 8.5%, 14.5%). Urinary cadmium concentrations followed an approximately log-normal distribution, with a median of 0.11 μg/L, an interquartile range (IQR) of 0.06–0.18 μg/L, and a range of 0.00–14.94 μg/L. Urinary cadmium concentrations by covariate level are listed in Table 1. Urinary cadmium concentrations were generally higher among non-Hispanic black and Mexican-American children than among white children, and higher among children from more impoverished households or households where the reference person had lower educational achievement (Table 1). Children with LD and children who participated in special education had higher median urinary cadmium levels, whereas children with ADHD had lower median urinary cadmium levels (Table 2).

**Table 1:** Urinary cadmium concentration by demographic and covariate category.^a^

Variable	n	Median urinary Cd [μg/L (IQR)]
Age(years)	
6-7	339	0.078 (0.038-0.139)
8-9	363	0.093 (0.050-0.151)
10-11	345	0.107 (0.058-0.180)
12-13	582	0.120 (0.062-0.199)
14-15	570	0.146 (0.071-0.220)
Urinary creatinine (mg/dL)	
8-78	541	0.052 (0.030-0.090)
79-117	551	0.090 (0.050-0.140)
118-174	553	0.130 (0.081-0.182)
175-614	554	0.201 (0.139-0.300)
Poverty income ratio	
0.00-0.82	514	0.130 (0.068-0.219)
0.83-1.51	502	0.110 (0.060-0.184)
1.52-2.99	507	0.105 (0.057-0.160)
3.00-5.00	507	0.090 (0.042-0.170)
Missing	169	0.124 (0.068-0.199)
Blood lead (μg/dL)	
0.2-0.8	489	0.095 (0.048-0.169)
0.9-1.2	469	0.111 (0.061-0.180)
1.3-1.9	532	0.110 (00.055-0.197)
2.0-57.1	496	0.122 (0.061-0.201)
Missing	213	0.100 (0.057-0.165)
Hemoglobin^b^	
Low	234	0.120 (0.061-0.207)
Normal/high	1,751	0.110 (0.057-0.182)
Missing	214	0.100 (0.057-0.165)
Race/ethnicity	
White	578	0.081 (0.040-0.150)
Black	712	0.137 (0.076-0.220)
Mexican	729	0.110 (0.060-0.172
Other Hispanic	90	0.096 (0.051-0.199)
Other race	90	0.113 (0.059-0.210)
Sex	
Male	1,144	0.110 (0.055-0.180)
Female	1,055	0.110 (0.060-0.183)
Serum cotinine (ng/mL)	
0.011-0.034	419	0.097 (0.050-0.170)
0.035-0.079	546	0.111 (0.059-0.188)
0.080-0.503	484	0.110 (0.053-0.188)
0.509-402.0	483	0.120 (0.070-0.193)
Missing	267	0.100 (0.058-0.165)
Smoker in the house	
Yes	477	0.112 (0.062-0.193)
No	1,697	0.110 (0.056-0.180)
Missing	25	0.109 (0.067-0.140)
Mother smoked while pregnant	
Yes	354	0.100 (0.052-0.169)
No	1,815	0.110 (0.058-0.185)
Missing	30	0.068 (0.063-0.177)
Mother's age at birth (years)	
15-20	521	0.128 (0.068-0.210)
21-24	545	0.114 (0.060-0.177)
25-29	528	0.096 (0.049-0.161)
30-44	550	0.103 (0.056-0.185)
Missing	55	0.119 (0.063-0.184)
Education of household reference person	
≤ 9th grade	306	0.125 (0.062-0.197)
≥ 9th no diploma	472	0.120 (0.070-0.200)
High school graduate/equivalent	531	0.104 (0.0.055-0.174)
Some college/associate arts degree	519	0.110 (0.054-0.180)
College graduate	295	0.081 (0.039-0.150)
Missing	76	0.118 (0.052-0.192)
^a^Values for the study population, not weighted for oversampling. bHemoglobin levels for age and sex based on information from Ahsan and Noether (2011): low hemoglobin, < 10th percentile; normal/high hemoglobin, ≥ 10th percentile.

**Table 2:** Urinary cadmium concentration by outcome status.^a^

Outcome	n (% total)	Median urinary Cd [μg/L (IQR)]
LD				
Yes		276 (12.6)		0.130 (0.063–0.213)
No		1,913 (87.0)		0.107 (0.056–0.179)
Missing/refused/don’t know		10 (0.5)		0.115 (0.070–0.210)
Special education				
Yes		231 (10.5)		0.130 (0.070–0.215)
No		1,965 (89.4)		0.108 (0.056–0.180)
Missing/refused/don’t know		3 (0.1)		0.059 (0.000–0.170)
ADHD				
Yes		198 (9.0)		0.100 (0.048–0.167)
No		1,997 (90.8)		0.110 (0.060–0.182)
Missing/refused/don’t know		4 (0.2)		0.121 (0.033–0.247)
aValues for the study population, not weighted for oversampling.				

Children in the two highest quartiles of urinary cadmium concentration had higher odds of LD and special education in both the creatinine-adjusted and fully adjusted analyses (Table 3). In contrast, children in the three highest quartiles of urinary cadmium concentration had lower odds of ADHD in the both the creatinine-adjusted and fully adjusted analyses (Table 3). When we compared children in the highest quartile of urine cadmium concentration with those in the lowest quartile, the fully adjusted odds ratios (ORs) were 3.21 (95% CI: 1.43, 7.17) for LD, 3.00 (95% CI: 1.12, 8.01) for special education, and 0.67 (95% CI: 0.28, 1.61) for ADHD. Adding a term for low hemoglobin level to the fully adjusted models had little effect on these ORs and did not change the significance conclusions (data not shown). ORs from core models adjusted only for urinary creatinine, age, sex, blood lead, smoker in the home, serum cotinine, prenatal smoke exposure (mother smoked while pregnant), and poverty income ratio were similar: 3.50 (95% CI: 1.56, 7.88) for LD, 2.66 (95% CI: 1.07, 6.63) for special education, and 0.65 (95% CI: 0.28, 1.51) for ADHD.

**Table 3:** ORs (95% CIs) for neurodevelopmental outcomes by quartile of urinary cadmium concentration.

Odds ratio (95% CI)		
Urinary Cadmium (μg/L)	Adjusted for creatinine onlya	Fully adjustedb
LD				
Quartile 1 (0.0000–0.0576)		1 (reference)		1 (reference)
Quartile 2 (0.0580–0.1097)		1.06 (0.63, 1.79)		0.98 (0.53, 1.79)
Quartile 3 (0.1100–0.1800)		1.56 (0.83, 2.91)		1.72 (0.88, 3.37)
Quartile 4 (0.1802–14.9400)		2.44 (1.09, 5.44)		3.21 (1.43, 7.17)
Special education				
Quartile 1 (0.0000–0.0576)		1 (reference)		1 (reference)
Quartile 2 (0.0580–0.1097)		1.51 (0.85, 2.69)		1.49 (0.80, 2.76)
Quartile 3 (0.1100–0.1800)		1.90 (0.77, 4.67)		2.15 (0.78, 5.93)
Quartile 4 (0.1802–14.9400)		2.41 (1.12, 5.18)		3.00 (1.12, 8.01)
ADHD				
Quartile 1 (0.0000–0.0576)		1 (reference)		1 (reference)
Quartile 2 (0.0580–0.1097)		0.58 (0.32, 1.04)		0.50 (0.25, 0.98)
Quartile 3 (0.1100–0.1800)		0.62 (0.32, 1.23)		0.52 (0.23, 1.19)
Quartile 4 (0.1802–14.9400)		0.58 (0.26, 1.31)		0.67 (0.28, 1.61)
aUrinary creatinine included as an independent covariate in the models. bCovariates included in the models: urinary creatinine, age, sex, blood lead, smoker in the home, serum cotinine, prenatal smoke exposure (mother smoked while pregnant), and poverty income ratio. LD model also included education level of household reference person. Special education model also included race/ethnicity, preschool attendance, and education level of household reference person. ADHD model also included race/ethnicity, mother’s age at birth, preschool attendance, and insurance coverage.				

When urinary cadmium was modeled with an ordinal tend variable (coded as 0, 1, 2, or 3 based on the quartile of exposure), the fully adjusted ORs for a one-quartile increase in urinary cadmium were 1.51 (95% CI: 1.14, 2.00) for LD, 1.44 (95% CI: 1.03, 2.01) for special education, and 0.84 (95% CI: 0.61, 1.16) for ADHD (Table 4). In sex-stratified analyses, the corresponding ORs for LD and special education were somewhat larger among males than among females [for LD, 1.75 (95% CI: 1.24, 2.46) vs. 1.24 (95% CI: 0.75, 2.04) and for special education, 1.73 (95% CI: 1.02, 2.92) vs. 1.31 (95% CI: 0.81, 2.13)]. In blood lead–stratified analyses, the corresponding OR for ADHD was 1.11 (95% CI: 0.65, 1.91) for those with lead levels below the median and 0.73 (95% CI: 0.52, 1.03) for those with lead above the median. However, these interactions were not significant (*p* = 0.71 for cadmium–sex in the LD analysis, *p* = 0.43 for cadmium–sex in the special education analysis, and *p* = 0.43 for cadmium–lead in the ADHD analysis), and none of the remaining cadmium–sex or cadmium–lead interaction terms were significant.

**Table 4:** Fully adjusted ORs (95% CIs) associated with a one-quartile increase in urinary cadmium concentration.

Stratum	LD	Special education	ADHD
Study population		1.51 (1.14, 2.00)		1.44 (1.03, 2.01)		0.84 (0.61, 1.16)
Below median lead		1.67 (1.09, 2.56)		1.74 (0.91, 3.30)		1.11 (0.65, 1.91)
Above median lead		1.39 (0.98, 1.95)		1.29 (0.83, 2.01)		0.73 (0.52, 1.03)
Female		1.24 (0.75, 2.04)		1.31 (0.81, 2.13)		0.91 (0.50, 1.69)
Male		1.75 (1.24, 2.46)		1.73 (1.02, 2.92)		0.79 (0.56, 1.11)


## Discussion

*LD and special education.* We observed that children in the highest quartile of urinary cadmium had significantly higher odds of both LD and special education when compared with those in the lowest quartile. A few prior studies have linked cadmium exposure with LD, and these studies relied on hair and blood samples to assess cadmium exposure. Two case–control studies demonstrated higher hair cadmium concentrations in children with LD (Capel et al. 1981; Pihl and Parkes 1977). A third study also found higher hair cadmium concentrations in children with LD, but the difference was statistically significant only for males (Ely et al. 1981). In that study, the authors considered the sexes separately but did not present the type of statistical evaluation for interaction that we report here. Interestingly, in our study the sex–cadmium interaction was not significant, but the effect estimate for urinary cadmium was larger among males. A fourth study reported no association between LD and blood cadmium in the 1999–2000 NHANES data (Lee et al. 2007). Cadmium accumulates in the kidney, and urinary cadmium concentration is considered to be a marker of cumulative exposure/body burden, whereas blood cadmium is thought to be a better indicator of recent exposure (Lauwerys et al. 1994). The different exposure metric used in the Lee et al. (2007) study may help explain the discrepancy in our findings.

In addition to higher odds of LD, we found that children in the highest quartile of urinary cadmium also had higher odds of special education placement. Special education is a “catch-all” outcome that likely involves a variety of neurocognitive and behavioral dysfunctions, including LDs such as reading difficulties, dyslexia, ADHD, and language/communication disorders, as well as behavior problems, psychiatric conditions, and perhaps some physical dysfunctions. The broad heterogeneous nature of both special education and LD as outcome measures prevents inferences about specific learning or cognitive domains. We are not aware of prior epidemiologic studies directly relating cadmium exposure and special education. However, any of the previously mentioned animal or human studies that link cadmium exposure to adverse neurobehavioral/neurocognitive or general health outcomes may be relevant here, because these outcomes could lead to special education placement.

There are data supporting the biological plausibility of cadmium exposure as a risk factor for LD and special education placement. For example, cadmium can inhibit the calcium flux required for neurotransmitter release (Hirning et al. 1988; Nation et al. 1989) and might thereby disrupt the neural communication required for synaptic network formation during development. Cadmium has also been shown to influence the proliferation and differentiation of neuroblasts in culture (Gulisano et al. 2009), and there is evidence that cadmium could indirectly affect the developing brain by disrupting thyroid hormone function (Iijima et al. 2007).

*ADHD.* Our findings for ADHD did not reach statistical significance, but the direction of the association suggests a possible decreased risk of ADHD diagnosis in children with urinary cadmium levels above the 25th percentile. There is only limited information from prior epidemiologic studies on cadmium exposure and ADHD/executive function. Lee et al. (2007) reported a nonsignificant trend of increasing odds of ADHD with increasing blood cadmium levels that was not present after adjustment for persistent organic pollutants based on an analysis of 1999–2000 NHANES data. As mentioned above, the different exposure metrics may partly explain the difference in our findings, because blood cadmium is a marker of recent exposure, whereas urine cadmium is a marker of chronic exposure (Lauwerys et al. 1994). Cao et al. (2009) reported a nonsignificant trend of increasing problem behavior scores at 7 years of age with increasing blood cadmium levels measured at 2 years of age, but there were no obvious trends with increasing blood cadmium in the attention/executive subdomains, the hyperactivity subdomain, or the ADHD index. The prospective approach is a strength of their study, but their unique population consisted solely of lead-poisoned children (blood lead levels of 20–44 μg/dL at enrollment), and the results may not be generalizable to non-lead-poisoned children.

Previous animal-based studies have demonstrated a variety of seemingly inconsistent effects of cadmium exposure on neurophysiology and activity levels. These findings include, for example, reduced exploratory activity and decreased time spent moving, but they also include hyperactivity, and evidence of changes in central nervous system dopamine and serotonin metabolism (Ali et al. 1986; Desi et al. 1998; Nation et al. 1989, 1990). These two neurotransmitter systems have been implicated in the etiology of ADHD (Faraone et al. 2005). The varied direction of effects on activity level seen in the animal literature might be related to differences in the timing of cadmium exposure during neurodevelopment, the presence of other uncontrolled variables, and/or the specific phenotype measured in each study. We lack detailed information on exposure timing, which makes it difficult to interpret the direction of the ORs for ADHD in the context of the animal literature (Andersen and Navalta 2004). If elevated cadmium exposure decreased activity levels, this may have made ADHD diagnosis less likely. It is also possible that cadmium may cause other neurocognitive dysfunctions that serve as competing risks to ADHD diagnosis (perhaps children with other diagnostic labels were less likely to receive an ADHD label), and the potential influence of chance should not be overlooked. Because these are cross-sectional data, the temporal relationship between exposure and outcome is not discernable, and it is possible that urinary cadmium concentration tended to decrease after ADHD diagnosis (e.g., children with ADHD may be more likely to have behaviors that decrease cadmium exposure, absorption, or excretion). In the lead-stratified analysis, we found evidence that the OR for ADHD is < 1 only among those with blood lead levels above the median. The interaction was not significant, but there is toxicologic evidence that cadmium exposure may attenuate lead-mediated increases in activity (Nation et al. 1990). Further research is needed to clarify these issues.

*Implications for cadmium risk assessments.* Previous cadmium risk assessments have considered renal effects to be the most sensitive end point of cadmium toxicity, and they identified urinary cadmium threshold levels that should protect against renal damage [EFSA 2009; World Health Organization/Food and Agriculture Organization of the United Nations (WHO/FAO) 2011]. Recent risk assessments by the European Food Safety Authority (EFSA 2009) and the WHO (WHO/FAO 2011) yielded urinary cadmium reference levels of 1 and 5.24 μg cadmium/g creatinine, respectively. When we excluded from the analyses the four study participants with urinary cadmium levels above the EFSA reference level, the associations between urinary cadmium and LD/special education were still evident (comparing the highest and lowest urinary cadmium quartiles: LD, OR = 3.25; 95% CI: 1.45, 7.28; special education, OR = 3.03; 95% CI: 1.14, 8.08). Thus, our work demonstrates associations with LD/special education at urinary cadmium levels below both the WHO and EFSA reference levels.

If these associations are replicated in other populations, then neurodevelopmental toxicity may be a sensitive end point to consider in future cadmium risk assessments. The EFSA and WHO risk assessments used toxicokinetic models to link creatinine-standardized urinary cadmium levels to dietary cadmium intake in order to estimate dietary intake standards (Amzal et al. 2009; EFSA 2009; WHO/FAO 2011). These toxicokinetic models were validated in adults (Amzal et al. 2009), but they are probably not appropriate to use among children, because exposure routes may differ (Weidenhamer et al. 2011) and because urinary creatinine concentration varies markedly with small increases in age among children (Barr et al. 2005). Recent work by Weidenhamer et al. (2011) suggests that mouthing or accidentally swallowing objects such as inexpensive jewelry may also contribute significantly to cadmium exposure in childhood. The extent to which these sources contributed to cadmium exposure in our population is unknown, but future risk assessments should not ignore the potential impact of non-food-based exposures or that current toxicokinetic models are not child specific.

*Strengths and limitations.* Exposure. The use of urinary cadmium as an exposure metric is a strength of this study. Urinary cadmium integrates exposure over many years (Lauwerys et al. 1994); thus, if brain development is sensitive to cadmium exposure in any of the time periods represented by this exposure metric, then this effect could be detected in our analysis. However, it is not possible to determine etiologically relevant time windows of exposure or to confirm the temporal sequence of exposure and outcomes based on the available data. A shorter time-course exposure metric such as blood cadmium (Lauwerys et al. 1994), in the context of a longitudinal prospective study that measures exposure and outcome at several time points, may be able to determine if the associations are driven by exposure that occurs in specific developmental windows.

Outcomes. We believe the diversity of outcomes evaluated is a strength of this study, because the combination of these three outcomes constitutes a screen for common neurodevelopmental dysfunctions. One limitation of these outcome measures is that they were derived from parent or proxy-respondent reports rather than neuropsychological evaluations. The use of ADHD treatments as an outcome would have likely identified only a subset of ADHD cases, resulting in low case numbers and analyses of limited power (Froehlich et al. 2007). Neuropsychiatric screening measures, such as the National Institute of Mental Health Diagnostic Interview Scale for Children that includes assessment of criteria for ADHD based on the 4th edition of the *Diagnostic and Statistical Manual of Mental Disorders* (CDC-NCHS 2006; Froehlich et al. 2009), would have been more objective, but these outcomes were not present in the publicly available NHANES data. Although outcomes were classified based on proxy-respondent reports, it is unlikely that outcome misclassification would be differential with respect to exposure unless the accuracy of reporting was related to unmeasured factors associated with cadmium exposure. Typically, nondifferential misclassification would be expected to bias associations toward the null (Rothman et al. 2008).

Confounding. The extensive covariate data available in NHANES, combined with the large sample size and high number of cases, allowed us to both evaluate and account for many potentially confounding variables. We sought to evaluate the association of cadmium exposure with the outcomes after accounting for other known correlates of the outcomes. We evaluated three sets of models: *a*) models adjusted only for creatinine, *b*) core models adjusted for primary potential confounders, and *c*) full models also adjusted for additional predictors of *a priori* interest. The conclusions from all three of these approaches were consistent. We further note that adjusting for iron deficiency using low hemoglobin had little effect on the results and does not alter the conclusions of this study (data not shown). As in any observational epidemiology study, we cannot rule out the possibility that confounding may have meaningfully affected our results. Potential sources of confounding in these analyses might include a lack of detailed information on the home environment (Bradley 1993) and parental psychopathology (Bellinger 2001).

Study design. The cross-sectional design of NHANES is a limitation of this study, because the temporal relationships between variables are not discernable. It is possible that higher cadmium exposure puts children at greater risk of LD/special education, but it is also possible that children with LD/special education have behaviors or prefer foods that increase their cadmium exposure. However, we are unaware of evidence supporting this reverse causation explanation.

The NHANES study design does offer strengths related to power and generalizability. To our knowledge, this is the largest study to evaluate associations between urinary cadmium and childhood learning/behavioral phenotypes. Because NHANES was designed to represent the noninstitutionalized U.S. population (CDC-NCHS 2010b), our findings should be generalizable to U.S. children 6–15 years of age.

## Conclusions

The results of this study are consistent with a growing body of evidence suggesting that elevated cadmium exposure may be associated with LD and special education. However, given the cross-sectional design and the nature of parent-reported outcomes, interpretations should be cautious. Prospective epidemiologic investigations and behavioral toxicology studies in animals could help to clarify if these associations are causal and if there are critical developmental windows for exposure. Prospective studies that consider co-exposures such as lead may also help reveal why the findings for LD/special education and ADHD differed. Given that the cadmium levels in this study represent typical exposure levels in U.S. children, our findings emphasize the need for further research into the potential neurodevelopmental effects of cadmium exposure.
